# External validation of SpineNetV2 on a comprehensive set of radiological features for grading lumbosacral disc pathologies

**DOI:** 10.1016/j.xnsj.2024.100564

**Published:** 2024-10-26

**Authors:** Alemu Sisay Nigru, Sergio Benini, Matteo Bonetti, Graziella Bragaglio, Michele Frigerio, Federico Maffezzoni, Riccardo Leonardi

**Affiliations:** a Department of Information Engineering, University of Brescia, via Branze 38, Brescia 25123, Italy; b Department of Clinical and Experimental Sciences, University of Brescia, Viale Europa, 11, Brescia 25123, Italy; c X-Ray Service s.r.l., Via Guglielmo Oberdan 126, Brescia, 25128, Italy; d Poliambulatorio Oberdan, Via Guglielmo Oberdan 126, Brescia, 25128, Italy

**Keywords:** AI for Medicine, Disc degeneration, Herniation, Low Back Pain, Spine MRI, SpineNetV2

## Abstract

**Background:**

In recent years, the integration of Artificial Intelligence (AI) models has revolutionized the diagnosis of Low Back Pain (LBP) and associated disc pathologies. Among these, SpineNetV2 stands out as a state-of-the-art, open-access model for detecting and grading various intervertebral disc pathologies. However, ensuring the reliability and applicability of AI models like SpineNetV2 is paramount. Rigorous validation is essential to guarantee their robustness and generalizability across diverse patient cohorts and imaging protocols.

**Methods:**

We conducted a retrospective analysis of MRI images of 1747 lumbosacral intervertebral discs (IVDs) from 353 patients (mean age, 54 ± 15.4 years, 44.5% female) with various spinal disorders, collected between September 2021 and February 2023 at X-Ray Service s.r.l. The SpineNetV2 system was used to grade 11 distinct lumbosacral disc pathologies, including Pfirrmann grading, disc narrowing, central canal stenosis, spondylolisthesis, (upper and lower) endplate defects, (upper and lower) marrow changes, (right and left) foraminal stenosis, and disc herniation, using T2-weighted sagittal MR images. Performance metrics included accuracy, balanced accuracy, precision, F1 score, Matthew's Correlation Coefficient, Brier Score Loss, Lin's concordance correlation coefficients, and Cohen's kappa coefficients. Two expert radiologists provide annotations for these discs. The evaluation of SpineNetV2′s grading is compared against expert radiologists' assessments.

**Results:**

SpineNetV2 demonstrated strong performance across various metrics, with high agreement scores (Cohen's Kappa, Lin's Concordance, and Matthew's Correlation Coefficient exceeding 0.7) for most pathologies. However, lower agreement was found for foraminal stenosis and disc herniation, underscoring the limitations of sagittal MR images for evaluating these conditions.

**Conclusions:**

This study highlights the importance of external validation, emphasizing the need for comprehensive assessments of deep learning models. SpineNetV2 exhibits promising results in predicting disc pathologies, with findings guiding further improvements. The open-source release of SpineNetV2 enables researchers to independently validate and extend the model's capabilities. This collaborative approach promotes innovation and accelerates the development of more reliable and comprehensive deep learning tools for the assessment of spine pathology.

## Introduction

Low back pain is a prominent cause of disability, accounting for 10.7% of the total Years Lived with Disability (YLDs) [28], where YLD is a metric that measures the impact of an illness on quality of life before resolution or death. This designation underscores the considerable challenge LBP poses to international health systems, as it casts a significant burden on healthcare [8,27]. The complexity of this challenge is multifaceted, as LBP is rooted in various potential causes, encompassing pathologies like disc narrowing, degeneration, canal stenosis, spondylolisthesis, modic changes, endplate defects, and herniation, each necessitating distinct diagnostic and treatment approaches [5].

Over the years, diagnostic tools commonly used for assessing intervertebral discs (IVDs) and their associated pathologies have included radiographs, X-rays, ultrasound, discography, computed tomography (CT), and magnetic resonance imaging (MRI). In recent times, considerable efforts have been directed towards creating and implementing AI applications in radiology which are focused on the spine and spinal cord [2,4,6,16,17,20,23,25]. These algorithms have wide-ranging purposes, including automatic detection and labeling of vertebral levels, automated grading of disc degenerative pathologies, detection and classification of spine trauma, identification of osseous lesions, and the assessment of cord pathology.

The integration of AI-based approaches, alongside traditional diagnostic tools, underscores a transformative shift in spinal imaging, with models like SpineNetV2 [30] aiming to enhance accuracy and efficiency in grading intervertebral disc (IVD) pathologies from MRI images. To effectively incorporate AI models into clinical practice, rigorous assessments of their robustness and generalizability are essential. This involves thorough evaluations across diverse imaging setups, patient cohorts, and protocols to ensure reliable performance in real-world settings.

In this study, we perform an external validation of SpineNetV2 [30], scrutinizing its grading outcomes against assessments provided by domain-specific radiologists for various disc pathologies. In particular, SpineNetV2, which has been trained specifically to analyze T2W lumbosacral spinal MRI images in the sagittal plane, represents the enhanced second version of the SpineNet system, incorporating various improvements compared to its initial iteration [14]. The MRI data used in this study are sourced from X-Ray Service s.r.l [31], a private clinic specialized in medical imaging services that provides over 10,000 imaging procedures annually. Our main objective is to expand the existing validations of SpineNetV2 model to encompass the entire set of radiological features it addresses. This strengthening of the scientific foundation supports the seamless integration of AI-assisted systems into clinical practice, particularly in the pivotal roles of aiding diagnosis and influencing treatment decisions [29,32].

To date, there have been 2 external validations [10,21] conducted on SpineNetV2, both characterized by limited validation scopes. Grob Alexandra, et al. [10] focused solely on validating 3 radiological features (Pfirrmann, Spondylolisthesis, and Central canal stenosis), while McSweeney Terence P. et al. [21] addressed 2 features (Pfirrmann and Modic changes) among the 11 features graded by SpineNetV2. In contrast, this study comprehensively evaluates SpineNetV2, on its complete set of 11 radiological considered features. Furthermore, our investigation aims to identify potential shortcomings in the SpineNetV2 software, serving as a basis for subsequent refinement and optimization. A dual emphasis on validation as well as weakness identification enables a deeper understanding of SpineNetV2’s applicability and possible optimization and enhancements, which emphasize its crucial role as a clinical decision AI-support tool.

## Materials and methods

In this section, we provide detailed information on the research process, the considered dataset, the annotation protocol adopted by the radiologists, the model under evaluation (SpineNetV2), the considered radiological features, and the evaluation metrics to be employed in assessing the model.

### Methods

This study was approved by the Institutional Review Board (IRB) of X-Ray Service s.r.l., ensuring that all ethical considerations were followed. Additionally, all images used in this study were obtained with formal consent from the patients involved.

**Fig. 1:**
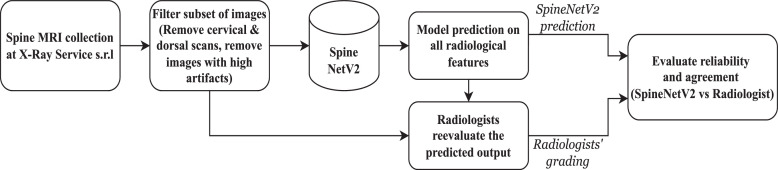
Summary of steps in the external validation process used in this study.

A visual summary outlining the validation process of this study is depicted in Fig. 1, offering a concise overview of the involved steps. Initially, spine MRI volumes gathered at X-Ray Service s.r.l. undergo a filtering process, which includes the removal of cervical and dorsal scans. The remaining scans are then analyzed by SpineNetV2, which provides predictions on all 11 radiological features. A performance drop was noticed in a few images, which were found to have high levels of artifacts, likely caused by patient movement during the scan. These images were subsequently excluded from the validation set. The outcomes of this automated grading by SpineNetV2 are presented to radiologists, who meticulously reevaluate the predicted outputs. The radiologists then provide their annotations, confirming or correcting the results based on their expertise. Finally, the evaluation process is completed by considering both the initial SpineNetV2 predictions and the radiologists’ grading annotations. This comprehensive approach allows for the assessment of reliability and agreement between SpineNetV2 and the radiologists, contributing valuable insights to the study.

### Dataset

A total of 1,615 spine MRI scans, collected between September 2021 and February 2023 at X-Ray Service s.r.l clinic [31] are considered. Table 1 summarizes the MRI protocol used in these scans. After excluding 1,145 thoracic and cervical images (as SpineNetV2 is trained only on lumbar discs), 89 images of patients below 14 years old, and 28 images with poor resolution and high artifacts, a total of 1,747 lumbosacral (L1-L2 to L5-S1) intervertebral discs (IVDs) from 353 MRI scans of patients (with a mean age of 54 ± 15.4 years, and 44.5% female) presenting diverse spinal disorders, are included for analysis.

**Table 1:** MRI acquisition protocol.

Orientation	Sagittal	Axial
Scanner manufacturer:	HITACHI Airis Vento	
Magnetic field strength (T)	0.3	
MR acquisition type	2D	
Scanning sequence	Spine Echo	
Repetition time (ms)	3135	5000
Echo time (ms)	108	110
Spacing between slices	4.7	5
Flip angle (degrees)	90	90

### SpineNetV2

SpineNetV2 [30] is a state-of-the-art open-source AI model which leverages a deep convolutional neural networkarchitecture for simultaneous grading of lumbar intervertebral disc (IVD) radiological features through multitask multiclass training. The training set of SpineNetV2 consists of MRIs sourced from patients participating in the *GENODISC* project [7], and from the *Oxford Whole Spine* (OWS) dataset. The *GENODISC* project accounts for 12,018 individual disc from MRI T2-weighted volumes from multiple centers in Europe. The *OWS* dataset instead, consists of 710 whole spine MR scans obtained with T1- and T2-weighted sequences across 196 patients, containing a range of degenerative changes including scoliosis, stenosis and collapsed vertebrae. The SpineNetV2 project also provides publicly accessible Python code with pretrained model parameters and weights [26] which we exploit to process the spine MRI collection at X-Ray Service s.r.l. clinic.

### Radiological features

**Fig. 2:**
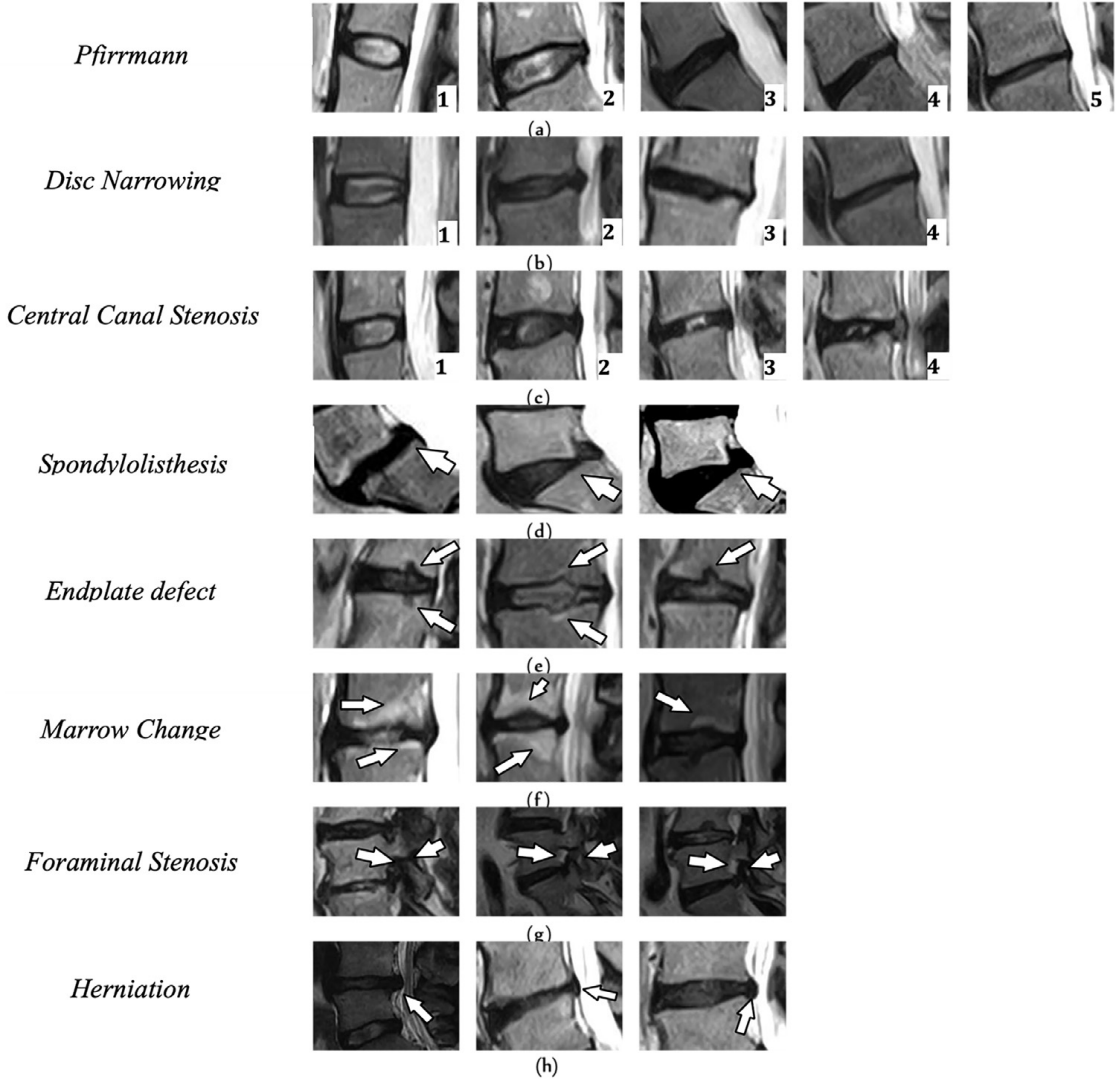
Examples of Sagittal T2w MRI slices for each radiological feature on all possible grading. (A): Pfirrmann grading [[1], [2], [3], [4], [5]], (B): Disc narrowing [[1], [2], [3], [4]], (C): Central canal stenosis [[1], [2], [3], [4]], (D): Spondylolisthesis [0–1], (E): End plate defect [0–1], (F): Marrow Change [0–1], (G): Foraminal Stenosis [0–1], (H): Herniation [0–1].

The current study focuses on all radiological features that are used in SpineNetV2 included *Pfirrmann grading, Disc narrowing, Central canal stenosis, Spondylolisthesis, Endplate defect* (*upper* + *lower*), *Marrow change* (*upper* + *lower*), *Foraminal stenosis* (*right* + *left*), and *Disc herniation*. Table 2 summarizes the grading and description of all considered radiological features, and Fig. 2 shows example MRI T2w sagittal slices for each radiological grading.

**Table 2:** A description of the radiological features considered in SpineNetV2.

Features	Descriptions	Range
Pfirrmann	A classification of disc degeneration based on disc signal heterogeneity, nucleus brightness, and disc height into 5 grades [24].	[1,...,5], where 1=normal, 5=severe degeneration.
Disc narrowing	A measurement of disc heights.	[1,...,4] where 1=normal, 4=extreme narrowing.
Central canal stenosis	The constriction of the central canal, in the region adjacent to each intervertebral disc.	[1,...,4] where 1=no stenosis, 4=severe canal stenosis.
Spondylolisthesis	A binary measure of vertebral slip.	[0-1], where 0=normal disc, 1=a Meyerding [18] grade of I, II, III, IV or V.
Endplate defects (Upper + Lower)	Any deformities of the endplate regions, both upper and lower, with respect to the intervertebral disc.	[0-1], where 0=No deformities 1=there is defect at lower or upper of the disc.
Marrow change (upper + lower)	A binary measure of the variations in visible signal on the upper or lower endplate extending into the vertebral body.	[0-1], where 0=no marrow change presented, 1=modic changes of type 1 or type 2 [22,13].
Foraminal stenosis (right + left)	A measure of narrowing in the neural foramen on the right or left side of the disc.	[0-1], where 0=no stenosis and 1=mild to severe stenosis [19].
Herniation	A binary measure of a condition where the centre of the IVD (nucleus pulposus) ruptures through its casing (annulus fibrosus).	[0-1], where 0=no herniation, 1=herniated disc.

### Radiologist grading protocol

Given the demonstrated potential of AI to augment human expertise, thereby mitigating the risk of prolonged assessments and overlooked diagnoses, particularly in cases involving mild or early stage pathologies, as highlighted in prior studies [1,3], we opt for an AI-assisted scenario.

In this approach, 2 expert radiologists, 1 with 17 years and the other with 38 years of experience, review the images alongside the predictions generated by SpineNetV2, addressing any disparities observed on all 11 radiological features.

As numerous studies highlight the positive impacts of AI-assisted systems eg, expediting radiology workflows, increasing inter-rater agreement and over-all accuracy [9,12,37], the specific choice of having humans validating or refining the AI-model predictions aims to minimize the possibility of overlooking subtle abnormalities, while also capitalizing on the efficiency afforded by automated predictions for timely grading.

In this scenario, 2 expert radiologists, 1 with 17 years and the other with 38 years of experience, review the images alongside the predictions generated by SpineNetV2, addressing any disparities observed on all 11 radiological features. By validating or refining the model predictions, we aim to minimize the possibility of overlooking subtle abnormalities, while also capitalizing on the efficiency afforded by automated predictions for timely grading.

The annotation protocol is organized in 2 steps. First, we assess the inter-rater agreement between the 2 radiologists who independently rated the initial 60 MRI cases, yielding compelling evidence of their consistency and reliability. Employing various agreement metrics on this initial set of 60 images, including *Brier Score Loss (BSL), Cohen's Kappa (κ), Lin's Concordance Correlation Coefficient* (LCCC), and *Matthews Correlation Coefficient* (MCC), we demonstrate high concordance and strong agreement across a diverse range of pathologies, as summarized in Table 3. Then, taking advantage of the observed strong agreement, and considering the scarce availability of a radiologist and the time-intensive nature of radiological evaluations, we streamline the data annotation process, by relying on the evaluation provided by a single radiologist for the remaining 293 patients.

**Table 3:** Inter-rater reliability and agreement evaluation of 2 expert radiologists on 289 discs acquired from 60 patients.

Metrics	Pfirrmann	Narrowing	Central Canal stenosis	Spondylolisthesis	Upper endplate defect	Lower endplate defect	Upper marrow change	Lower marrow change	Right foraminal stenosis	Left foraminal stenosis	Herniation
BSL	0.055	0.055	0.0138	0.007	0.031	0.059	0.066	0.083	0.038	0.035	0.045
*κ*	0.823	0.838	0.851	0.930	0.835	0.734	0.769	0.795	0.890	0.901	0.900
LCCC	0.973	0.966	1.00	1.00	0.908	0.828	0.842	0.797	0.99	0.980	0.962
MCC	0.823	0.839	0.851	0.930	0.835	0.736	0.77	0.695	0.890	0.901	0.900

BSL, Brier score loss; κ, Cohen's kappa; LCCC, Lin's concordance correlation coefficient; MCC, Mathews correlation coefficient.

### Agreement and reliability metrics

In assessing the external validation of a deep learning model, inter-rater agreement and reliability metrics [11] play a pivotal role, offering a comprehensive insight into the concordance between the model predictions and radiologists’ annotations. This study employs the following metrics to evaluate the level of agreement and assess the model's performance:

1. **Accuracy:** measures the overall correctness of predictions:
  $$Accuracy=\;\frac{Number\;of\;Correct\;Predictions}{Total\;Number\;of\;Predictions};$$
2. **Precision**: evaluates the proportion of true positives among all predicted positives:

$Precision=\;\frac{TP}{TP+FP};$
where *TP* are True Positives, and *FP* are False Positives.

3. **Recall**: evaluates the proportion of true positives among all actual positive samples in the dataset. Recall can also be called *Sensitivity*, or *True positive rate*:
  $$Recall=\;\frac{TP}{TP+FN};$$
  where *FN* are False Negatives.
4. **F1 score:** is the harmonic mean of precision and recall:
  $$\begin{array}{ccc} F1 & = & \frac{2}{Recall^{-1}+Precison^{-1}}=2*\frac{Recall*Precision}{Recall+Precision}\\ & = & \frac{2TP}{2TP+FP+FN}; \end{array}$$
5. **Balanced accuracy:** adjusts accuracy by averaging the sensitivity and specificity of the model:
  $$\begin{array}{ccc} Balanced\;accuracy & = & \;\frac{Sensitivity+Specificity}{2}\\ & = & \frac{1}{2}\left(\frac{TP}{TP+FN}+\;\frac{TN}{TN+FP}\right); \end{array}$$
  where: *TN* refers to True Negatives, *Sensitivity* refers to the *‘true positive rate’* which represents the percentage of positive cases that the model correctly identifies. *Specificity*, on the other hand, denotes the *‘true negative rate’* indicating the percentage of negative cases that the model correctly identifies.
6. **Matthew's Correlation Coefficient (MCC):** captures the correlation between predicted and observed binary classifications:
  $$MCC=\;\frac{TP*NP+FP*FN}{\sqrt{\left(\text{TP}\;+\;\text{FP}\right)*\;\left(\text{TP}\;+\;\text{FN}\right)*\;\left(\text{TN}\;+\;\text{FP}\right)*\;\left(\text{TN}\;+\;\text{FN}\right)}};$$
7. **Lin's concordance correlation coefficient (CCC):** is the concordance between a model prediction (Y) and a gold standard test or measurement (X):
  $$CCC\;=\;\frac{2C_{xy}}{C_{xx}+C_{yy}+{\left(\mu_{x}-\mu_{y}\right)}^{2}};$$
  where:
  • *C_XY_* is the sample covariance between predicted outcomes (Y) and actual values (X);
  • *C_XX_* and *C_YY_* are the sample variances of X and Y, respectively;
  • *µ_x_* and *µ_y_* are the means of the actual values and predicted outcomes, respectively.
8. **Brier Score Loss**: is a measure of the mean square difference between the predicted and actual probability:
  $$Barier\;Score\;Loss\;=\;\frac{1}{N}\sum_{i=1}^{N}{\left(P_{i}-O_{i}\right)}^{2};$$
  where N is the number of observations, *P_i_* is the predicted probability, and *O_i_* is the observed outcome (0 or 1).
9. **Cohen's Kappa** is a measure of inter-rater reliability or the agreement between 2 dependent categorical samples:
  $$\kappa =\frac{P_{o}-P_{e}}{1-P_{e}};$$
  where:
  • *P_o_* is the Observed Agreement: $P_{o}=\frac{TP+TN}{N}$
  • *P_e_* is the Expected Agreement: $P_{e}=\frac{\left(TP+FP)*(TP+FN)+(TN+FN)*(TN+FP\right)}{N^{2}}$, on a total of *N* observations.

## Results

The evaluation of the SpineNetV2 performance versus radiologist grade, on a total of 1,747 intravertebral discs and across various pathologies, reveals notable level of agreement between the specialist and the grading offered by the AI system.

Regarding *Pfirrmann grading*, the model demonstrates an accuracy of 79.6%, achieving balanced accuracy of 79.4%, with precision, recall, and F1 score all at 79.9%. In the case of *Narrowing*, the model exhibits a commendable accuracy of 86.7%, along with balanced accuracy of 84.9%, and precision, recall, and F1 score all exceeding 86%. *Central canal stenosis* evaluation showcases an exceptional accuracy at 97.1%, albeit with a slightly lower balanced accuracy of 77.9%, while the precision, recall, and F1 scores maintain parity at 97.1%.

*Spondylolisthesis*, reflecting critical pathology, demonstrates an outstanding accuracy at 98.3%, with balanced accuracy reaching 95.2%, and precision, recall and F1 score above 98%. Robust performance is also observed for *Endplate defects* (upper and lower) and *Marrow changes* (upper, lower) with accuracies of 94.8%, 94.2%, 94.0% and 93.1%, balanced accuracy scores of 84.9%, 86.3%, 89.5% and 89.2% and average precision, recall and F1 scores of 94.6%, 94.1%, 94.15% and 93.4%, respectively. The model performance on detecting *Foraminal stenosis* (left and right) has an accuracy of 85.2% and 85.4% with balanced accuracy scores of 70.2% and 69.1%, and average scores of precision, recall and F1 of 84.36% and 84.5%, respectively. For detecting *Disc herniation*, the model returns accuracy of 79% with balanced accuracy score of 75.9% and average score of precision, recall and F1 score of 79.3%.

In addition to evaluating the model classification performance, we rigorously assess its reliability and agreement with human raters through a set of diverse metrics. The *Brier Score Loss*, a measure of predictive accuracy, exhibits variations across pathologies, ranging from 0.014 to 0.210, highlighting specific areas of the model's proficiency and potential for improvement. *Cohen's Kappa* coefficients, elucidating the degree of agreement between the model and human rater, demonstrates concordance scores spanning from 0.457 to 0.799. *Lin's Concordance Correlation Coefficient* (LCCC), a metric reflecting both precision and accuracy, consistently presents high values, emphasizing the model capability to maintain robust concordance with human rating, ranging from 0.530 to 0.972 across different pathologies. The *Matthews Correlation Coefficient* (MCC), considering true and false positives and negatives, ranges from 0.483 to 0.799 across different pathologies, providing subtle insights into the model's overall performance. These results are summarized in Table 4.

**Table 4:** Primary evaluation metrics of the SpineNetV2’s capabilities and performance across several pathologies, as well as the assessment of its reliability and agreement metrics with human raters.

Metrics	Pfirrmann	Narrowing	Central Canal Stenosis	Spondylolisthesis	Upper endplate Defect	Lower Endplate Defect	Upper Marrow Change	Lower Marrow Change	Right Foraminal Stenosis	Left Foraminal Stenosis	Herniation
Accuracy	0.796	0.867	0.971	0.983	0.948	0.942	0.940	0.931	0.852	0.854	0.790
BAS	0.794	0.849	0.779	0.988	0.849	0.863	0.895	0.892	0.702	0.691	0.759
Precision	0.799	0.869	0.971	0.983	0.946	0.940	0.943	0.937	0.841	0.843	0.810
Recall	0.796	0.868	0.971	0.983	0.948	0.942	0.940	0.931	0.852	0.854	0.790
F1 Score	0.796	0.868	0.971	0.985	0.947	0.941	0.941	0.933	0.838	0.838	0.779
BSL	0.081	0.066	0.014	0.016	0.052	0.058	0.060	0.070	0.148	0.146	0.210
*κ*	0.738	0.799	0.749	0.705	0.732	0.745	0.756	0.731	0.473	0.457	0.546
LCCC	0.952	0.972	0.932	0.745	0.880	0.920	0.896	0.873	0.542	0.530	0.630
MCC	0.738	0.799	0.749	0.721	0.733	0.745	0.757	0.734	0.494	0.483	0.576

BAS, balanced accuracy score; BSL, Brier score loss; κ, Cohen's kappa; LCCC, Lin's concordance correlation coefficient; MCC, Mathews correlation coefficient.

The bolded results indicate the best score observed for each metric across all graded pathologies.

**Fig. 3:**
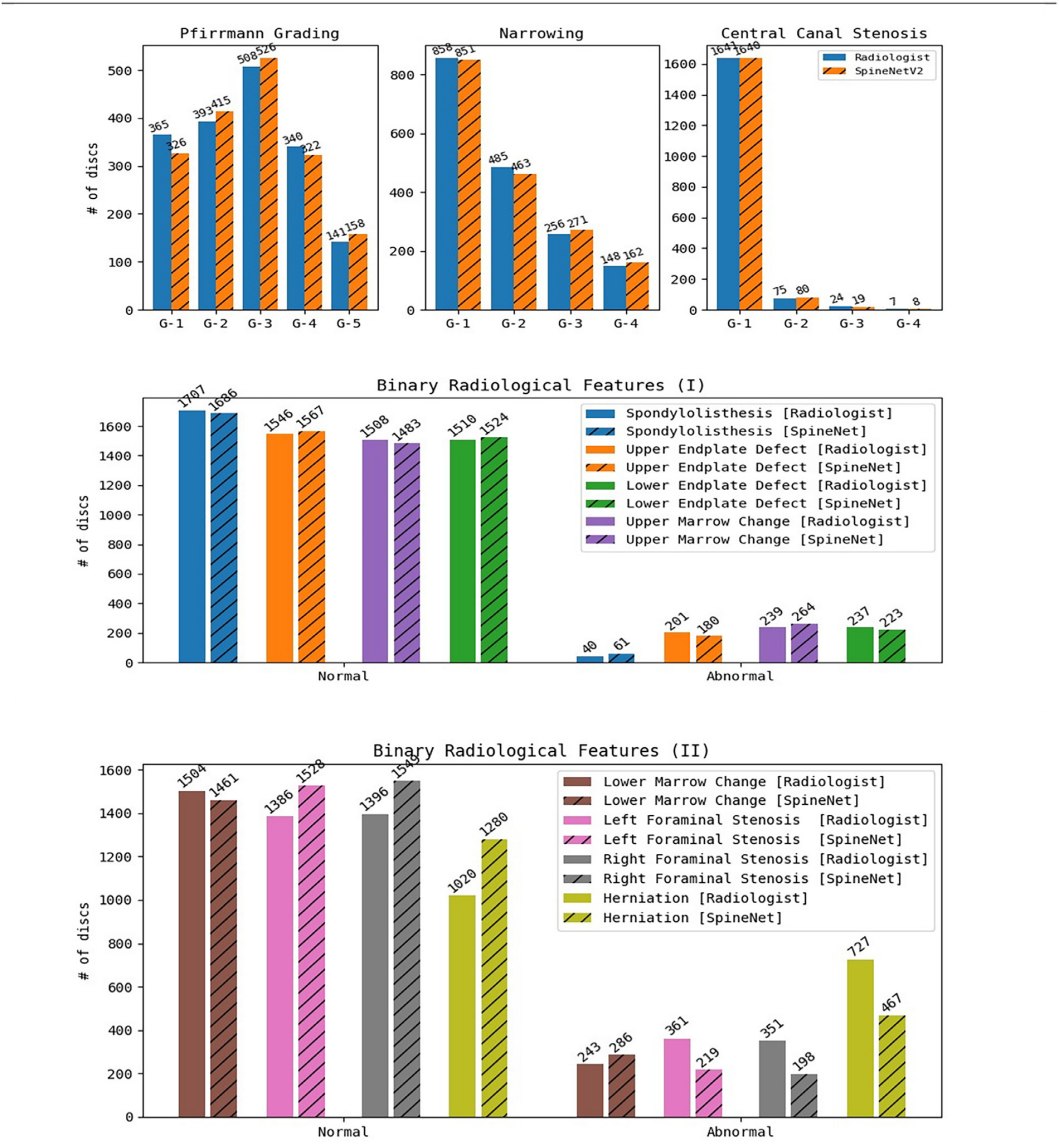
Histograms depicting the distribution of the radiological grades given by the specialist and by SpineNetV2 of all patients and all vertebral levels. On the vertical axis the absolute count is depicted, and on the horizontal axis the severity grades are given.

**Fig. 4:**
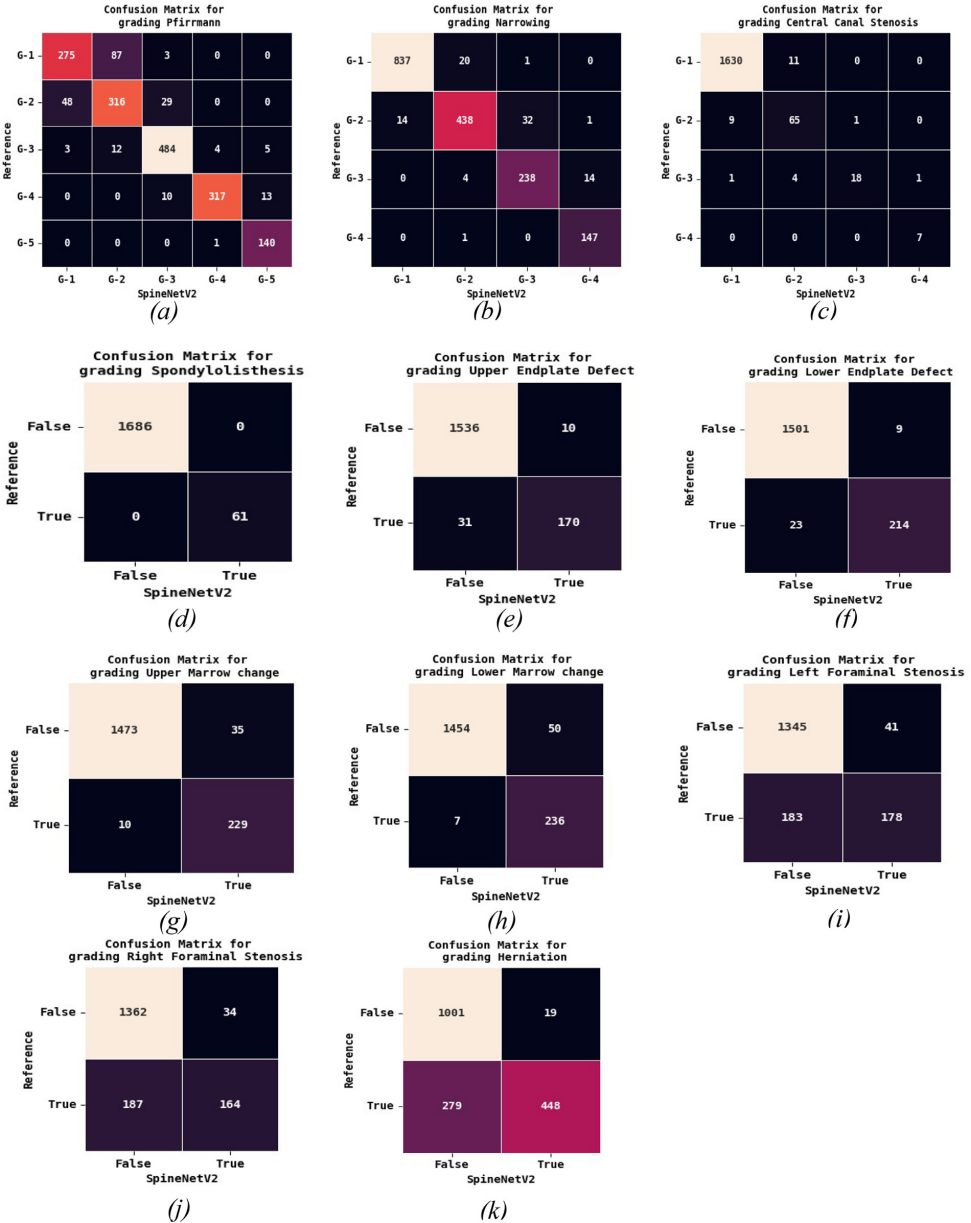
Confusion Matrices: Reference Radiologist vs. SpineNetV2 grading: (A): Pfirrmann grading, (B): Narrowing, (C): Central Canal Stenosis, (D): Spondylolisthesis, (E): Upper endplate defect, (F): Lower endplate defect, (G): Upper marrow change, (H): Lower marrow change, (I) Left foraminal stenosis, (J): Right foraminal stenosis, (K): Herniation.

Furthermore, the distribution of radiological grades given by the radiologist and by the SpineNetV2 for all radiological features are visually depicted in Fig. 3. To further provide an exhaustive evaluation of SpineNetV2 grading against the reference radiologist grading across all features, we present a detailed confusion matrix for each radiological feature in Fig. 4.

## Discussions

In the evolving field of AI for medical imaging, particularly in spine pathology grading, there have been notable efforts aimed at grading disc-related pathologies from MRI data. However, these existing studies have limitations:

Liawrungrueang et al. [33], focused solely on Pfirrmann grading, utilizing sagittal T2-weighted MRI. While it used an open-source dataset, it lacks publicly available code and models for external validation.

Lim et al. [34], developed a deep learning model to assist radiologists in interpreting spinal canal, lateral recess, and neural foraminal stenoses from lumbar spine MRI scans. The study used sagittal T2 for foraminal stenosis and axial T2 for spinal canal and lateral recess evaluation. However, it does not provide open-source code or models for validation, and no cross-validation of the results was performed.

Shinde et al. [35], employed a semi-automated system for grading disc degeneration, focusing on a single radiological feature. As with the other studies, there are no open-source models or code for independent verification.

Lu et al. [36] proposed a CNN model for grading central canal and foraminal stenosis, leveraging both axial and sagittal imaging series, and applied report-derived labels to the corresponding imaging segments. Eventhough it used both axial and sagittal series, it solely focused on central canal and foraminal stenosis. They performed comparative analysis of those 2 pathologies with SpineNetV2 and reported as their model outperforms the SpineNetV2 as they used both axial and sagittal series.

In light of the limitations seen in these studies such as restricted scope, lack of open-source validation, and absence of comprehensive feature analysis the SpineNetV2 model stands out as a more robust, publicly accessible, and clinically viable AI solution that deserves to be validated externally.

Regarding *Pfirrman grading*, the findings from this study provide strong evidence supporting the use of SpineNetV2 for Pfirrmann grading of lumbar MRIs. Our results show a moderate agreement (kappa value of 0.738) and a class balanced accuracy score of 79.4%, outperforming what measured in previous studies such as Winsdor et al. (70.9%) [30]. Additionally, the current study demonstrates a substantial concordance with Lin's concordance correlation coefficient of 0.95%, further confirming the reliability and consistency of SpineNetV2 in comparison to human assessments (reported in Jamaludin et al. [14] with kappa=0.701). The distinction between Pfirrmann Grades 1 and 2 is challenging due to their shared characteristics of a clear distinction between nucleus and annulus, as well as a normal height of the intervertebral disc. Consequently, radiologists often classify both grades as normal. This difficulty is also reflected in SpineNetV2, as shown in the confusion matrix (Fig. 4A). The model misclassifies grade 2 cases as grade 1 (48 cases) and grade 1 cases as grade 2 (87 cases), underscoring the challenge in accurately differentiating between these grades. This misclassification error is not treated as a serious concern and is anticipated given the inherent difficulty in distinguishing between these 2 grades.

The grading of *Disc narrowing* shows a strong agreement and correlation between the radiologist rating and the SpineNetV2 prediction (kappa=0.799). This high score suggests that *Disc narrowing* is easily detectable by the model, possibly due to its clear association with the height of the discs. Additionally, Jamaludin et al. [15] also reported a strong concordance score of LCCC=0.89 for *Disc narrowing* grading, further supporting the reliability of this feature. While there exists a strong concordance between the model and radiologist, a notable number of misclassified errors emerge when differentiating between grade 1 and 2. However, the misclassified discrepancies depicted in the confusion matrix (Fig. 4B) for these grades are not deemed significant concerns, given that both grades are typically regarded as falling within the normal range.

The grading of *Central canal stenosis* shows a moderate agreement between the radiologist rating and SpineNetV2 grading, with a kappa value of 0.749. Furthermore, there is a strong correlation between the 2 methods, as indicated by the LCCC value of 0.932. The *Mathew's correlation coefficient* (MCCC) further supports their correlation, with a value of 0.749. Similarly, McSweeney et al. [21] also reports a moderate agreement with a *kappa value* of 0.6 and a *correlation MCC* value of 0.57.

**Fig. 5:**
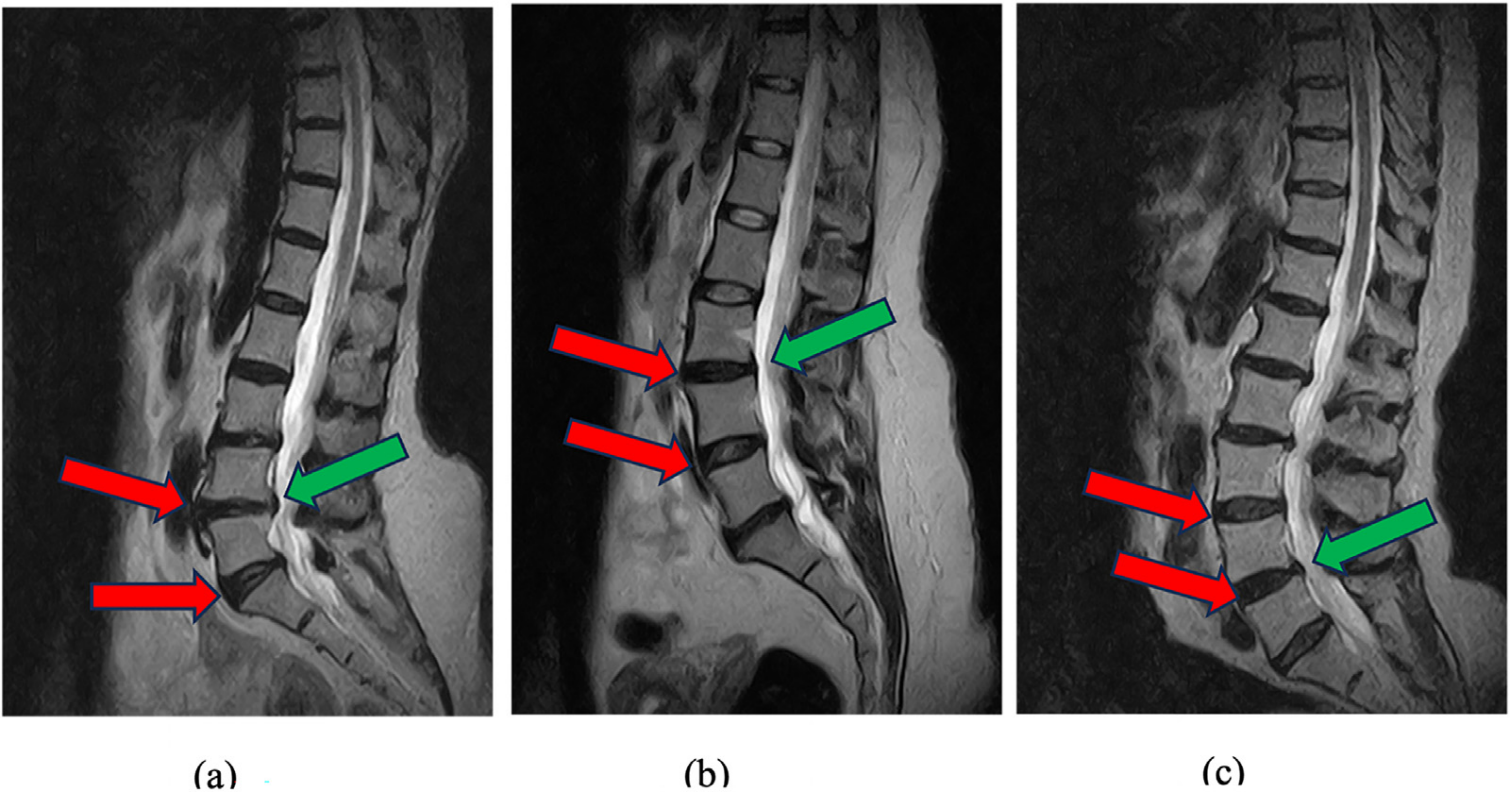
Lumbar T2 magnetic resonance imaging mid-sagittal slices showing cases of disagreement on Spondylolisthesis grade between SpineNetV2 and radiologist. (A), L4-L5 and L5-S1 intervertebral discs graded as grade 1 by SpineNetV2 (red arrow) but only L4-L5 disc is grade 1 by radiologist (green arrow). (B), L3-L4 and L4-L5 intervertebral discs graded as grade 1 by SpineNetV2 (red arrow) but only L3-L4 disc is grade 1 by radiologist (green arrow). (C), L3-L4 and L4-L5 intervertebral discs graded as grade 1 by SpineNetV2 (red arrow) but only L4-L5 is grade 1 by radiologist (green arrow).

SpineNetV2 system for grading *Spondylolisthesis*, although Winsdor et al. [30] reports that it is designed to perform a binary grading (grade 0 and 1) for spondylolisthesis, the model occasionally produces a third class (grade 2) for certain images. To make a direct comparison with other works, we merge grades 1 and 2 into a single pathological class. We then observe a moderate agreement (kappa=0.705) and correlation (LCCC=0.74) between the radiologist and SpineNetV2. This agreement is higher if compared to the ones reported by McSweeney et al. [21] and Jamaludin et al. [15] (kappa=0.63 for both). When grading these characteristics, we observe that for some patients, the system predicts the neighboring disc to be also positive whereas *Spondylolisthesis* was present in only one of the discs. Fig. 5 illustrates some examples of *Spondylolisthesis* on a single disc that is classified as positive by the radiologist, whereas SpineNetV2 classifies 2 neighboring discs as both positive.

The SpinenetV2 model demonstrates excellent performance in grading both *Upper and Lower Endplate defects*, achieving an accuracy and F1 score of over 90%. Furthermore, there is a reasonable level of agreement between the model ratings and the assessments made by the radiologist, as indicated by kappa values of 0.73 and 0.74 respectively. In addition, the LCCC values of 0.88 and 0.920 respectively further confirm this agreement. It is worth noting that only Jamaludin et al. [15] have conducted validation on this particular feature, and their findings show a lower level of agreement (0.49 and 0.55).

In the case of grading *Marrow changes*, we observe a high level of agreement between the ratings of upper and lower marrow changes, with kappa values of 0.756 and 0.731, respectively. This is consistent with the findings of Jamaludin et al. [15], who report an agreement of 0.63 and 0.62 between the radiologist and SpineNetV2. Furthermore, McSweeney et al. [21] report the same level of agreement found in our study, even though they did not explicitly mention the upper and lower marrow changes (we assume that the reported results are averages of the upper and lower marrow changes [*κ*=0*.*74]).

For grading left and right *Foraminal stenosis*, we obtained a lower agreement and correlation scores compared with the other features. We record weak agreement and poor concordance (kappa 0.47 and 0.45, concordance 0.54 and 0.53) between radiologist and SpineNetV2. The same lower performance as exhibited by low correlation scores are also observed when grading herniation (*κ*=0*.*54 and *LCCC*=0*.*63). These degradations can be explained to the fact that *Foraminal stenosis* and *Herniation* can be better visualized using both axial and sagittal MRI images, as these views provide a more comprehensive view of the spine. Using only sagittal images, on the other hand, these lack sufficient evidence for the accurate assessment of these pathologies. However, the lack of other validation studies on these features prevent us to confirm this hypothesis, for which other studies are needed.

Despite the promising results of this study, several limitations should be acknowledged. Firstly, the reliance on sagittal T2-weighted MRI images may restrict the accuracy of assessing certain pathologies, such as foraminal stenosis and herniation, which could benefit from axial imaging for better visualization. Secondly, while SpineNetV2 shows strong performance for various grading tasks, there were instances of misclassification, particularly regarding Spondylolisthesis, where the model occasionally labeled adjacent segments as positive even when only 1 disc exhibited the condition. Lastly, the absence of external validation for certain radiological features limits the ability to independently verify the model's robustness. Future research would benefit from addressing these limitations by incorporating diverse imaging modalities and enhancing the explainability of model predictions to improve its applicability in clinical practice.

## Conclusion

Nowadays, the need to perform an independent external validation of deep learning models in medical imaging is imperative in order to assess performance of robustness and generalizability across a variety of datasets and imaging protocols from an unbiased perspective. In this context, SpineNetV2, a state-of-the-art open-source model, designed for grading disc pathologies from sagittal view MRI images, requires scrutiny in various clinical settings. Two previous works [21,10] attempted external validation on SpineNetV2, showcasing the potential usability of the model. The current study builds upon this foundation, expanding the scope of the investigated features, thus emphasizing the importance of openly accessible models. Previous studies carried out external validations but were limited in scope, evaluating only a subset of pathologies (*Pfirrmann, Spondylolisthesis, Modic Change, and Central Canal Stenosis*). Our work addresses this gap by comprehensively validating SpineNetV2 across all 11 disc pathologies evaluated by SpineNetV2, recognizing the importance of conducting extensive evaluation for robust clinical applicability. Such inclusion in our external validation contributes to a more complete understanding of the model capabilities, offering insights into its reliability and potential limitations. The results reveal robust performance in various metrics, including accuracy, balanced accuracy, precision, and the F1 score. Notably, high agreement scores (*Cohen's Kappa, Lin's Concordance Correlation Coefficient*, and *Mathew's Correlation Coefficient* exceeding 0.7) are observed for the majority of pathologies. However, discrepancies arose in (right and left) *Foraminal stenosis* and *Herniation*, highlighting the limitations of relying solely on sagittal view MRI images for these specific pathologies. The overall high agreement scores reinforce SpineNetV2 potential for accurate pathology prediction for the rest of radiological features, emphasizing its clinical utility in diverse settings. The challenges observed in *Foraminal stenosis* and *Herniation* underscore the need to consider both the axial and the sagittal views for a more comprehensive assessment. This limitation can offer valuable insights for future model enhancements.

## Declaration of competing interest

The authors declare that there are no conflicts of interest regarding the publication of this manuscript.
